# The role of immune profile in predicting outcomes in cancer patients treated with immunotherapy

**DOI:** 10.3389/fimmu.2022.974087

**Published:** 2022-11-03

**Authors:** Andrea Botticelli, Giulia Pomati, Alessio Cirillo, Simone Scagnoli, Simona Pisegna, Antonella Chiavassa, Ernesto Rossi, Giovanni Schinzari, Giampaolo Tortora, Francesca Romana Di Pietro, Bruna Cerbelli, Alessandra Di Filippo, Sasan Amirhassankhani, Alessandro Scala, Ilaria Grazia Zizzari, Enrico Cortesi, Silverio Tomao, Marianna Nuti, Silvia Mezi, Paolo Marchetti

**Affiliations:** ^1^ Department of Radiological, Oncological and Pathological Science, Sapienza University of Rome, Rome, Italy; ^2^ Department of Molecular Medicine, Sapienza University of Rome, Rome, Italy; ^3^ Department of Medical and Surgical Sciences and Translational Medicine, University of Rome “Sapienza”, Rome, Italy; ^4^ Medical Oncology, Fondazione Policlinico Universitario Agostino Gemelli Istituti di Ricovero e Cura a Carattere Scientifico (IRCSS), Rome, Italy; ^5^ Medical Oncology, Universitá Cattolica del Sacro Cuore, Rome, Italy; ^6^ Istituto Dermopatico dell’Immacolata, Rome, Italy; ^7^ Department of Medico-Surgical Sciences and Biotechnology, Polo Pontino, Sapienza University, Rome, Italy; ^8^ Laboratory of Tumor Immunology and Cell Therapy, Department of Experimental Medicine, Policlinico Umberto I, University of Rome “Sapienza”, Rome, Italy; ^9^ Department of Urology, S. Orsola-Malpighi Hospital University of Bologna, Via Palagi, Bologna, Italy

**Keywords:** immunotherapy, tumor biomarker, cytokines, chemokines, soluble immune check-points

## Abstract

**Background:**

Despite the efficacy of immunotherapy, only a small percentage of patients achieves a long-term benefit in terms of overall survival. The aim of this study was to define an immune profile predicting the response to immune checkpoint inhibitors (ICIs).

**Methods:**

Patients with advanced solid tumors, who underwent ICI treatment were enrolled in this prospective study. Blood samples were collected at the baseline. Thirteen soluble immune checkpoints, 3 soluble adhesion molecules, 5 chemokines and 11 cytokines were analyzed. The results were associated with oncological outcomes.

**Results:**

Regardless of tumor type, patients with values of sTIM3, IFNα, IFNγ, IL1β, IL1α, IL12p70, MIP1β, IL13, sCD28, sGITR, sPDL1, IL10 and TNFα below the median had longer overall survival (p<0.05). By using cluster analysis and grouping the patients according to the trend of the molecules, two clusters were found. Cluster A had a significantly higher mean progression free survival (Cluster A=11.9 months vs Cluster B=3.5 months, p<0.01), a higher percentage of disease stability (Cluster A=34.5% vs. Cluster B=0%, p<0.05) and a lower percentage of disease progression (Cluster A=55.2% vs. Cluster B = 94.4%, p=0.04).

**Conclusion:**

The combined evaluation of soluble molecules, rather than a single circulating factor, may be more suitable to represent the fitness of the immune system status in each patient and could allow to identify two different prognostic and predictive outcome profiles.

## 1 Background

Immune checkpoint inhibitors (ICIs), a class of drugs targeting the inhibitory immune checkpoint receptors, have revolutionized clinical practice in oncology, demonstrating a significant improvement in progression-free survival (PFS) and overall survival (OS) in many types of cancer (1). The ability of immune cells to recognize, kill and control tumor cells has a strong impact in tumor progression (2). On the other hand, tumor immune-evasion mechanisms are mainly responsible for determining the failure of therapeutic strategies (3). Several studies have demonstrated that, in a portion of patients, ICIs could overcome tumor immune evasion, inducing a durable immune response against tumors (4). Thus, immunotherapy has become the standard of care for several cancer including advanced melanoma (5), non-small cell lung cancer (NSCLC) (6, 7), metastatic renal cell carcinoma (RCC) (8) and locally advanced and metastatic head and neck squamous cell carcinoma (HNSCC) (9). Even in uveal melanoma (UM), although considered a different clinical and biological entity from cutaneous melanoma, immunotherapy has become an important first line option (10). Nevertheless, some patients fail to respond to ICIs or become resistant during treatment. Early detection of intrinsically resistant patients is a crucial issue in clinical practice, as it could prevent immunotherapy failure (11–13). New, robust data are required to develop and validate molecular and genetic predictive biomarkers of ICIs resistance. In recent years, research focused on sampling soluble immune checkpoint (sIC), circulating molecules of adhesion, as well as cytokines and chemokines (14, 15).

Tumor cells employ several mechanisms to escape the control of the immune system. Among these processes, tumor microenvironment associated soluble factors and/or surface-bound molecules are mostly responsible for dysfunctional activity of the immune system (16). Recent results suggest that the concentration of these sICs is lower in patients benefitting from immunotherapy, with a potential role in predicting time to treatment failure (14, 17).

Soluble programmed death-ligand 1 (PD-L1) can inhibit the activation of either infiltrating or circulating T cells by means of PD-1/PD-L1 pathway (18, 19). CD-137, released as soluble form, negatively regulates the activation of T cells, blocking the interaction between T cells and antigen presenting cells (APCs) (20). These soluble factors, produced by alternative splicing or through proteolytic shedding of extracellular region of the cellular membrane can impede efficacy of ICI antibodies acting as decoy from the drug.

In this study a large spectrum of circulating molecules was analyzed, including soluble immune check-points, cytokines/chemokines and adhesion molecules, in patients with advanced/metastatic solid tumors before anti-PD-1 treatment. Focus was put on the differences in immune systems at baseline, trying to create a soluble immune profile (SIP) which could preemptively identify immunotherapy responder or non-responder patients.

## 2 Materials and methods

### 2.1 Clinical data

This prospective, multicentric study included patients with advanced or metastatic solid tumors including NSCLC, UM, RCC and HNSCC, who started immunotherapy between January 2017 and December 2020. Patients aged 18 years or older were included, with histologically confirmed solid tumors with advanced and/or metastatic disease, eligible for immunotherapy. Patients with Eastern Cooperative Oncology Group performance status (ECOG PS) ≤ 2 with adequate bone marrow, renal and liver function, fit for immunotherapy and able to provide a signed informed consent were included. Patients with ECOG PS >2 and patients with absolute contraindications to immunotherapy were excluded from the study. Baseline staging was performed according to the TNM system (AJCC 8th edition), with contrast-enhanced computed tomography (CT) and contrast-enhanced magnetic resonance imaging (MRI) based on clinical judgement. Age, sex, baseline, ECOG PS, previous treatments received and tumor histology data were collected.

ICI treatment was administered according to the standard schedule approved for each primary tumor and line of treatment. Nivolumab was administered at the standard dose of 240 mg intravenously at 2-weeks interval and pembrolizumab at the standard dose of 200 mg intravenously at 3-weeks interval. Imaging assessment was performed after 12 weeks, or earlier in case of evident clinical disease progression. Tumor response was assessed using immune-related Response Evaluation Criteria in Solid Tumors (i-RECIST) and classified as complete response (CR), partial response (PR), stable disease (SD), and progressive disease (PD).

Progression free survival (PFS) was defined as the time from the first administration of ICIs until the first progression or in-treatment death. Overall survival (OS) was defined as the time from patient registration, or treatment commencement, to death from any cause or last follow up available.

Data were collected anonymously into a specific database. Protocol approval from Local Ethics Committee was obtained [CE 4421].

### 2.2 Samples collection

Peripheral blood samples were drawn from 81 patients with advanced/metastatic solid tumors before starting immunotherapy with anti-PD-1 agents (Nivolumab or Pembrolizumab). Peripheral blood samples were collected at baseline (T0) in red top collection tubes to allow blood to clot. After centrifugation at 1,500 x g for 10 minutes, serum samples were collected and stored at - 80°C until use. Immunomonitoring analyses were performed evaluating soluble circulating molecules.

### 2.3 Circulating soluble molecules

The immune profile was studied as an ensemble of 11 inflammatory cytokines, 5 chemokines, 3 soluble adhesion molecules and 13 soluble immune checkpoint molecules (**Table 1**) through a multiplex assay using the ProcartaPlex Human Inflammation Panel (catalog number EPX200-12185-901) and the Human Immuno-Oncology Checkpoint 14-plex ProcartaPlex Panel 1 (catalog number EPX14A-15803-901) (eBioscience) and evaluating the following circulating immune molecules: sE-Selectin; ICAM-1/CD54; IFN alpha; IFN gamma; IL-1 alpha; IL-1 beta; IL-4; IL-6; IL-8; IL-10; IL-12p70; IL-13; IL-17A/CTLA-8; IP-10/CXCL10; MCP-1/CCL2; MIP-1alpha/CCL3; MIP-1 beta/CCL4; sP-Selectin; TNF alpha, CD137, CTLA4, PD1, PDL1, PDL2, TIM3, LAG3, GITR, HVEM, BTLA, CD80, CD27 and CD28. For each patient, an amount of 50 µl of serum was used and added to a 96 well plate together with a mixture of magnetic beads coated with an antibody, according to the manufacturer’s instructions. After that, a biotinylated detection antibody was added to the plate and then bound to Phycoerythrin (PE)-conjugated streptavidin. Samples were measured in single using the Luminex 200 platform (BioPlex, Bio-Rad). Data, expressed in pg/ml, were analyzed using Bio-Plex Manager Software. Subsequently to the evaluation two soluble molecules, i.e. IDO and GM-CSF, were excluded from the analysis. GM-CSF was not considered because the instrument didn’t detect its serum value for the majority of patients. The exclusion of IDO, on the other hand, is due to poor reliability of the multiplex method, infact its activity evaluation is preferentially performed by high-performance liquid chromatography tandem mass spectrometry, in which is evaluated the concentration of kynurenine and tryptophan.

**Table 1:** Soluble immune molecules: Characteristics and function.

Soluble molecules	Class of molecules	Cell source	Ligands	Main function	Type of action
**sCD137**	sIC	PBMCs	CD137L	Inhibits CD137/CD137L binding	Inhibitory
**sPD1**	sIC	PBMCs	PDL1/PDL2	Blocks PD1/PDL1 interactions	Activatory
**sPDL1**	sIC	Mature DCs	PD1	Binds PD1 and inhibits T cells response	Inhibitory
**sPDL2**	sIC	Tumor exosomes, alternatively activated macrophages	PD1	Unknown	Unknown
**sCTLA4**	sIC	Monocytes, immature DCs, regulatory T cells	CD80/CD86	Inhibits T cell responses	Inhibitory
**sTIM3**	sIC	Activated lympocytes	Tim3-L	Unknown	Unknown
**sLAG3**	sIC	Activated and exhausted CD4^+^, CD8^+^ T cells, regulatory T cells	Unknown	Unknown	Unknown
**sGITR**	sIC	Macrophages and regulatory T cells	GITRL	Unknown	Unknown
**sCD27**	sIC	Activated lymphocytes	CD70	Unknown	Unknown
**sCD28**	sIC	T cells	CD80/CD86	Inhibits T cells activity and counteracts anti-PD1 activity	Inhibitory
**sBTLA**	IC sIC	T cells, B cells, dendritic cells and myeloid cell	HVEM	Unknown	Unknown
**sHVEM**	sIC	T cells, B cells, natural killer cells, monocytes, neutrophils and dendritic cells		Unknown	Unknown
**sCD80**	sIC	Unstimulated monocytes and B cells	CTLA4/CD28	Unknown	Unknown
**sICAM-1**	Molecoles of adhesion	B and T lymphocytes Endothelial cells	LFA-1	binding the transmembrane receptor, antagonises leukocyte recruitment	Inhibitory
**sE-selectin**	Molecules of adhesion	Endhotelial cells	Carbohydrate ligands on tumor cells, sialyl Lewis-X	-Enhance angiogenis - Upregulation of ICAM-1 on tumor cells	unknown
**sP-selectin**	Molecules of adhesion	Endhotelial cells	PSGL-1, sialyl Lewis-X	-leukocyte recruitment -metastatisation -masking of tumor cells by binding to platelets	immune evasion
**MCP1**	chemokine	macrophage monocytes	CCR2 CCR4	-leucocyte recruitment	proinflammatory
**MIP1 α MIP1 β**	chemokine	Macrophages Hematopoietic cells	CCR1 CCR5	-granulocyte degranulation -production of pro-inflammatory cytokines -Promote cronic inflammation	proinflammatory
**IP10**	chemokine	Monocytes Endotelial cells fibroblast	CXCR3	Leucocytes recruitment	proinflammatory
**INFα**	Cytokine	DC Macrophages NK cells Macrophages Endothelial cells Fibroblasts	INFαR1/2	-NK activation -Cells B proliferation -Possible suppression of -Treg cells -Antiviral activity -Enhances MHC expression	proinflammatory immune-activation
**INFϒ**	Cytokine	Lymphocytes T (th1) CD8 and NK	INFγR1/2	-activation of macrophages -activation of Th1 responses -potential antigen presentation to T lymphocytes -induces apoptosis of tumor cells and reduces VEGF -increases expression of IDO	immunoactivating/ possible immunosuppressive activity)
**TNFa**	Cytokine	Macrophages NK T cells	TNFR1 TNFR2	-pro-inflammatory activity -stimulates cell proliferation and survival -induction of apoptosis -implicated in resistance to antiPD1 drugs	Immune-activation/ pro-inflammatory
**IL1α**	Cytokine	DC Macrophages Neutrophils Endothelial cells fibroblast	IL1R1 IL1R2	-production of acute phase proteins -stimulates TNFa pathway -implicated in fever, sepsis and inflammation	Immune activation Pro-inflammatory
**IL1β**	Cytokine	DC Macrophages Neutrophils Endothelial cells fibroblast	IL1R1	-production of acute phase protein -implicated in fever -induces differantiation of lymphocytes Th17	Immune activation Pro-inflammatory
**IL4**	Cytokine	T cells Mast cells	IL4-Rα	-activation of Th2 immune response -cell growth/activation	Pro-inflammatory
**IL6**	Cytokine	Macrophages Endothelial cells T cells	IL6Rα	-B lymphocyte proliferation and antibody response -production of prostaglandins and acute phase proteins -antagonises Treg -anti-inflammatory action through inhibition of TNFa and induction of IL10	Pro-inflammary/anti-inflammatory
**IL8**	Chemokine	Macrophages Endothelial cells Platelets	CXCR1 CXCR2	- chemotaxis -powers phagocytosis -ability to mediate infiltration of MDSCs into the tumor environment	Immuneactivation/ Immune-evasion
**IL10**	Cytokine	Macrophages Treg cells B cells Mast cells Th2 Tcells	IL10Rα IL10Rβ	-downregulation of Th1 cytokines -inhibits CD4 T cell activity -suppresses expression of costimulatory molecules -increases survival of B lymphocytes -blocks secretion of proinflammatory cytokines	Antinflammatory/ Possible immunostimulating anti-tumor activity
**IL12p70**	Cytokine	Macrophages DC	IL12Rb1 IL12Rb2	-activation of Th1 responses -powers CD8 and NK T-cell activity -Increases INFa production by T cells -suppresses Treg proliferation and angiogenesis	Immune activation
**IL13**	Cytokyne	T CD4 Cells CD8 cells NK Eosinophils Mast cells	IL13Rα1 IL13Rα2	-involved in Th2 immune responses -potential expression of adhesion molecules on endothelial cells -activation of magrophages and production of TGFb	Proinflammatory
**IL17A**	Cytokine	Lymphocytes TCD4 Th17	IL17Rα	-induces IL6 and chemokines production - promotes recruitment of MDSCs into the tumor bed	Proinflammatory

DC, dendritic cells; IL, interleukin; IFN, interferon; TNF, tumor necrosis factor; MCP, monocyte chemoattractant protein; MIP, macrophage inflammatory protein; IP, interferon induced protein; pd-l1, programmed death ligand 1; CTLA-4, Cytotoxic T-Lymphocyte Antigen 4; TIM3, T-cell immunoglobulin domain and mucin domain 3, LAG3, lymphocyte Activating 3; B- and T-lymphocyte attenuator; HVEM, Her-pesvirus entry mediator; ICAM-1, Intercellular Adhesion Molecule 1.

### 2.4 Statistical analysis

Statistical evaluation was performed using the statistical package SPSS Release Version 21.0 (SPSS Inc., Chicago, IL). Statistical significance cut-off level was set for p < 0.05. All tests of significance were two tailed. Continuous data were shown as means and categorical data were shown as frequencies (percentiles). Differences between continuous data were evaluated using the nonparametric Mann–Whitney U-test. In univariate analysis, the non-parametric Mann-Whitney U-test (two groups) was first used to compare soluble molecules continuous values in subjects with a given type of cancer; then, each variable of interest was dichotomized (as under the respective median or above the median) to study the OS or PFS in the two groups thus obtained. In addition, each variable of interest was dichotomized (as below or above the median value) to study the proportion of subjects with OS < or >12Mo in the two groups thus obtained. Categorical variables were compared between groups using the Chi squared test. Pearson’s Chi squared test or Fisher’s exact test (used for two-by-two contingency tables with less than 50 cases) were used to assess if paired observations on two variables, expressed in a contingency table were independent of each other. Multiple logistic regression was performed for the clinical variables with dichotomous scores to investigate whether associations between OS and soluble immune checkpoint were present after simultaneously adjusting for other variables of interest. Separate modelling was performed for each condition including all molecules, in addition to sex and age. P values <0.05 were considered significant. Since survival and prognosis varies widely by primary tumor type, 12 months was used as the cut-off value to assess the association between molecule concentration and survival, as it is similar to the median OS of the study population. Moreover, this value could be suitable, in our opinion, in discriminating the slice of patients primarily resistant to immunotherapy (21–23).

A time to event analysis was performed using non-parametric Kaplan-Meier (KM) product limit survival estimates, and differences between KM survival curves were analyzed using the Mantel-Haenszel log-rank test. Relatedness of soluble molecules was tested by applying unsupervised Eisen’s hierarchical cluster methods (24) to the data set, encompassing immune molecules across all samples and using as agglomeration rule the average linkage clustering as implemented in the Genesis soft-ware (25). Unsupervised clustering involved the sorting of both soluble ICs and cytokines/chemokines/adhesion molecules values. The soluble molecules tree was computed on the basis of a full data set and the distances between samples were computed by using Pearson correlation as similarity measures. Each square in the heat-map represents the higher value (red), equal value (black) or lower level (green) of signal of any given test-ed soluble molecule for each tested subject.

The color intensity of every single square in the heatmap is directly associated with the measured concentration in pg/ml. Interpretation of the heat-map generated by the software could be performed either visually, where clustering distinct soluble factors tends to give more homogeneous areas, or by taking into consideration the higher or lower level of dendrograms on the patient side of the graph.

## 3 Results

### 3.1 Patients

Eighty-one metastatic patients treated with anti PD-1 agent were enrolled in this study: 22 patients with UM, 10 patients with RCC, 13 with HNSCC, and 36 with NSCLC. Baseline clinical–pathological characteristics of patients are summarized in **Table 2**. All 10 patients in the RCC group had clear cell carcinoma and all 13 HNSCCs had squamous histology. Fifty-one patients were male (63%), 30 patients were female (37%). The mean age was 51 ± 9 years. All patients were treated with anti-PD-1 agents (nivolumab and pembrolizumab): 25 patients in a first-line, 56 patients in a second- or subsequent-line setting.

**Table 2:** Clinicopathological characteristics.

Parameter	N (%)	UM	RCC	HNSCC	NSCLC
Total	81	22	10	13	36
Age years(mean, range)	51 ± 9	67 ± 10	56 ± 10	63 ± 9	65 ± 9
Gender					
Male	51 (63%)	11	8	9	25
Female	30 (37%)	11	2	4	11
Pembrolizumab	25	22	–	–	3
Nivolumab	56	–	10	13	33
I line	25	22	–	–	3
II/subsequent line	56	–	10	13	33

UM, uveal melanoma; RCC, renal cell carcinoma; HNSCC, head and neck; squamous cell carcinoma; NSCLC, non small cell lung cancer.

### 3.2 Outcomes

Median OS was 27.4 ± 25:2 months in UM, 49.2 ± 20.7 months in RCC, 18.5 ± 11.5 months in HNSCC, and 24.8 ± 24 months in NSCLC. Median OS was significantly lower in HNSCC than in the RCC group (p<0.05). Median PFS was 9 ± 10.8 months in UM, 17.6 ± 16.23 months in RCC, 4.9 ± 5 months in HNSCC, and 12.6 ± 14.6 months in NSCLC.

### 3.3 Soluble profile by type of cancer

#### 3.3.1 Serum value of sICs

Mean values of each sIC in UM, RCC, HNSCC and NSCLC are shown in **Table S1**. There is a wide heterogeneity of soluble ICs serum levels between cancers. The same table shows moreover the statistically significant differences between sICs value means in the tumor subgroups. In NSCLC, sCD27 had the highest values. A similar trend was noted for sCD137, sHVEM and sLAG3 levels. sHVEM values were higher in RCC when compared to UM and HNSCC. On the other hand, RCC showed the lowest levels of sPDL2, which had its greater values sampled in HNSCC and NSCLC patients. HNSCC patients had the highest values of sCD80 and sCTLA4, and the lowest levels of sPDL1.

#### 3.3.2 Serum value of soluble adhesion molecules

**Table S2** shows the main value of each soluble adhesion molecule and the statistically significant differences in the comparison between pairs of tumor subgroups. The highest sICAM-1 values were found in the HNSCC group and their lowest ones in the UM group. Similarly, the highest sP-selectin values were found in HNSCC patients, and RCC showed lower values when compared to HNSCC and NSCLC. The sE-selectin value was higher in HNSCC and NSCLC when compared to UM and RCC groups.

#### 3.3.3 Serum value of cytokines/chemokines

**Table S3** shows the mean serum value of cytokines and chemokines in each cancer subgroup with a highlight on their statistically significant differences. Cytokines and chemokines levels were the highest in NSCLC patients, except for IL17A values. Mean value of IL17A was significantly higher in HNSCC compared to other types of cancer. In UM IP10 had the lowest values compared to all the other types of cancer, even though lower values of the other cytokines and chemokines were noted, when compared to HNSCC and NSCLC. Lower concentrations of IFNγ, MCP1, MIP1 β and TNFα were found in RCC than in HNSCC and NSCLC. IL10 values were found to be lower in the HNSCC group than in RCC and NSCLC.

### 3.4 Soluble molecules and oncological outcomes

#### 3.4.1 Differences between patients with OS below and above 12 months

There are significant differences in the mean values of several cytokines and chemokines (IFNα, IFNγ, IL10, IL12p70, IL13, IL1α, IL1 β, IL4, IL8, MCP1, MIP1α, MIP1β and TNFα) between the OS < 12 months group and the OS > 12 months group (**Table S4**). The concentrations of all of these soluble factors were significantly lower in patients with OS longer than 12 months. Multiple logistic regression analysis, considering simultaneously all the molecules studied together with age and sex, showed a significant relationship between OS and IL1α levels (p: 0.037 ORa= 0.151, 95% CI= 0.025-0.893).

#### 3.4.2 Multiple Soluble ICs and cytokines/chemokines correlation with OS and PFS

**Figure 1:**
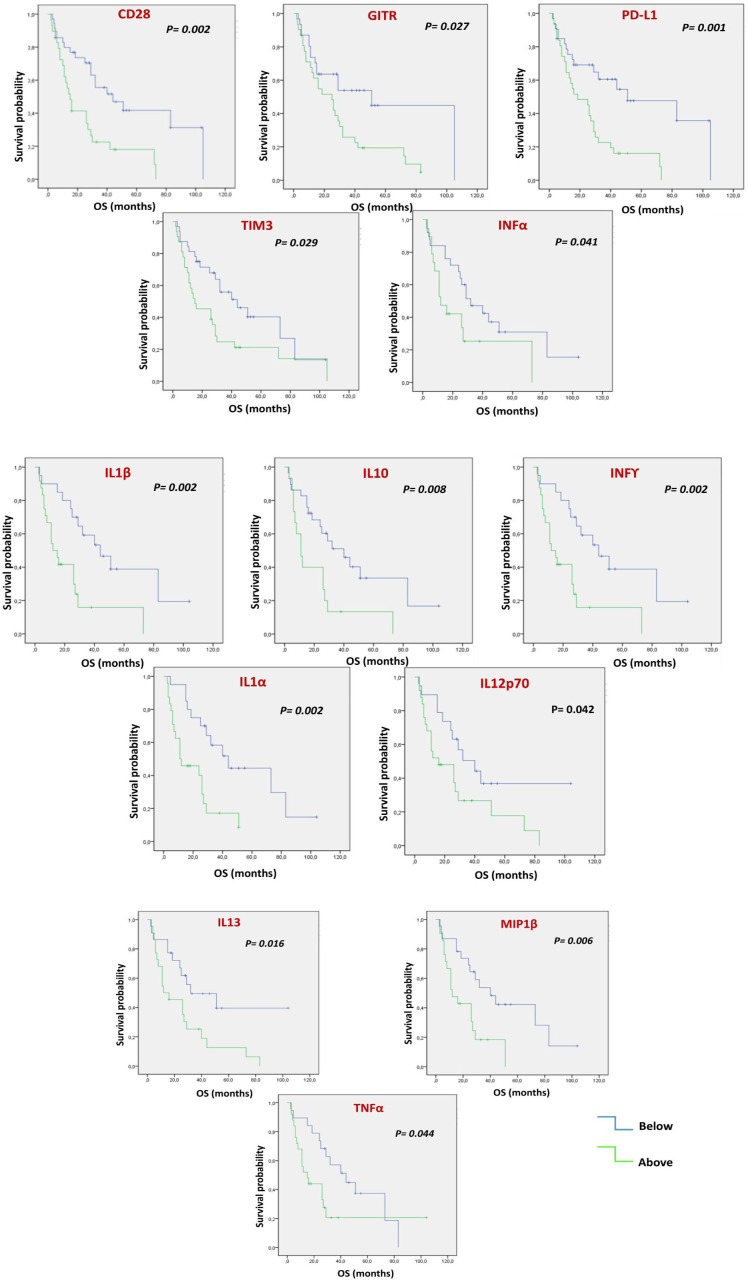
Multiple Soluble ICs and cytokines/chemokines are correlated with OS. Each value of soluble factor, regardless of cancer type, was dichotomized as under the median or above the median. Kaplan-Meier evaluation showed that low values of soluble CD28, GITR, PDL1, TIM3, INFα, INFγ, IL1β, IL10, IL1α, IIL12p70, IL13, MIP1β and TNFα were associated with better OS (p<0.05).

**Figure 2:**
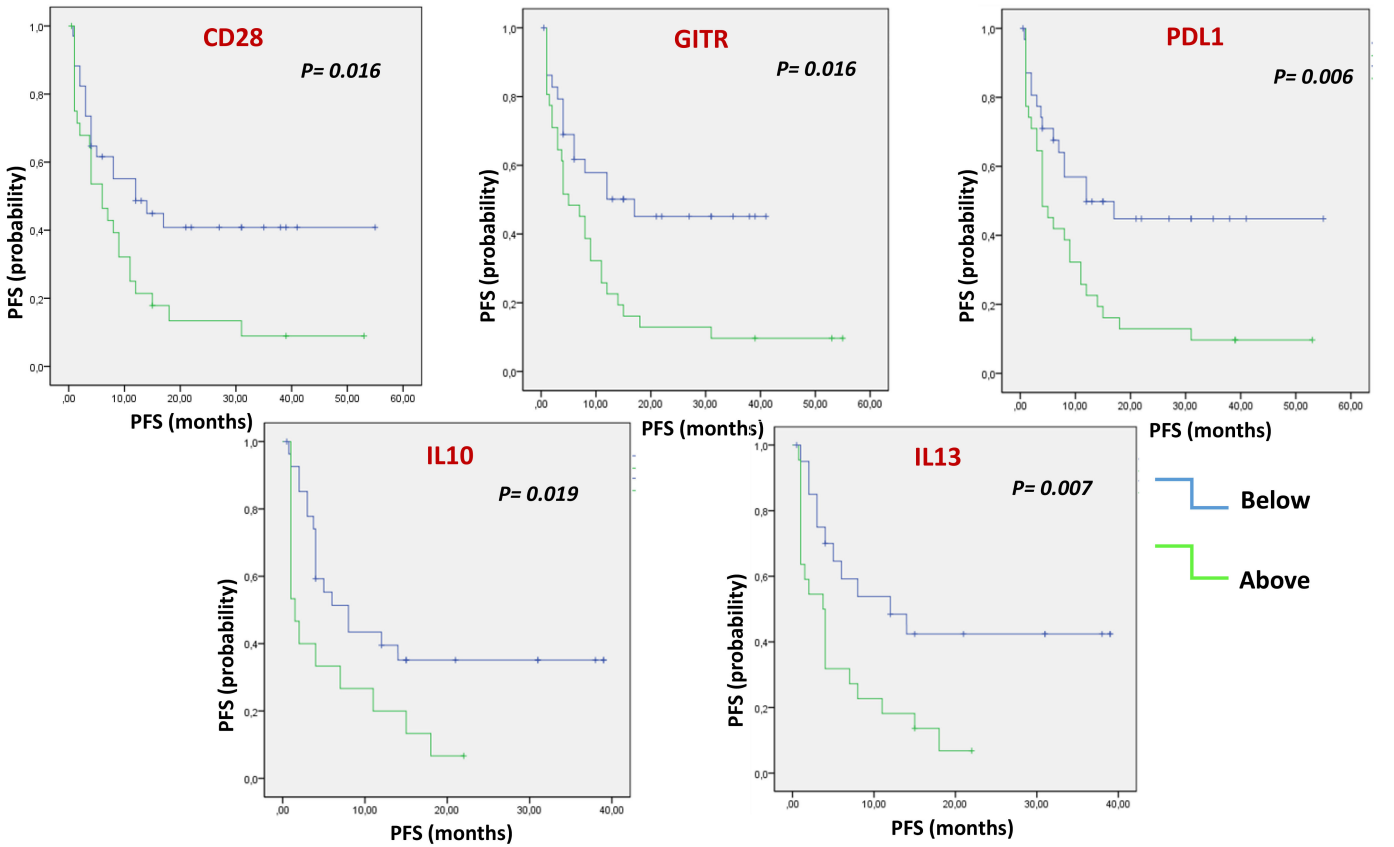
Multiple Soluble ICs and cytokines/chemokines are correlated with PFS. Kaplan-Meier evaluation showed that, dichotomizing values of soluble factors under or above the median, low levels of soluble CD28, GITR, PDL1, IL10 and IL13, were associated with longer PFS (p<0.05).

Each soluble factor was dichotomized based on the median value found (**Figure 1** and **Table S5**). After Kaplan-Meier evaluation, significantly longer OS was found in patients with low levels of sCD28, sGITR, sPDL1, sTIM3, IFNα, IFNγ, IL1β, IL10, IL1α, IL12p70, IL13, MIP1β and TNFα. Furthermore, each soluble factor was also dichotomized based on the median value found in relation to PFS (**Figure 2** and **Table S6**). Significantly longer PFS in patients with low levels of sCD28, sGITR, sPDL1, IL10 and IL13 were found.

### 3.5 Comprehensive prognostic and predictive immune profile

**Figure 3:**
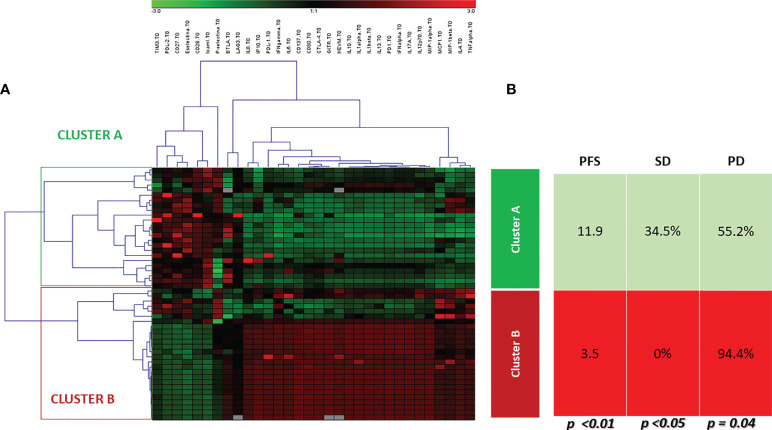
Unsupervised hierarchical cluster analysis. **(A)** The heat-map of cluster analysis. Soluble molecule tested are listed in the top of the figure. The unsupervised hierarchical cluster analysis identified 2 distinct clusters of patients based on the soluble immune profile associated with a different oncological outcome: Cluster A (green box) and Cluster B (red box). The color intensity of every single square in the heat-map is directly associated with the measured concentration in pg/ml. Each square in heat-map represents the higher value (red), equal value (black) or lower level (green) of signal of any given tested soluble molecules for each tested patient, **(B)** Oncological outcomes were reported for each cluster. Cluster A was associated with longer PFS and higher SD rate than Cluster B (11.9 months vs. 3.5 months, and 34.5% vs.0, respectively).

**Figure 4:**
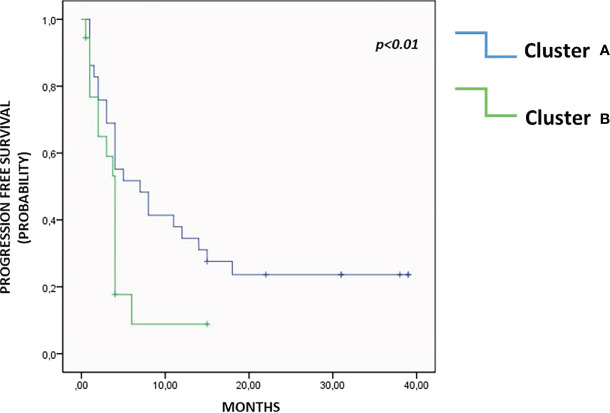
Progression free survival. As highlighted in Kaplan Meier curves, patients in Cluster A showed a significantly longer PFS than patients in Cluster B, 11.9 months vs. 3.5 months, p<0.01.

Unsupervised hierarchical cluster analysis, performed for those patients with all available soluble evaluation, identified two distinct groups of patients (Cluster A and B) based on soluble molecules serum levels, prior to ICI therapy (**Figure 3**). The distribution of cancer types varied between the two clusters. Cluster A included 9 UMs, 6 RCCs, 12 NSCLCs and 2 HNSCCs cases, while cluster B included 7 UMs, 2 RCCs, 2 NSCLCs and 10 HNSCCs. No significant differences were shown between the two clusters for rate of patients in the II-line setting (68.9% cluster A and 66.6% cluster B, p=0.251) and platinum-refractory patients (48.2% in Cluster A vs. 57.1% in cluster B, p=0.535). The first group represented patients with high concentration of the soluble checkpoints sTIM3, sPDL2, sCD27, sCD28 and adhesion molecules. The second group, in addition to the remaining soluble ICs (sPD1, sPDL1, sCD137, sCD80, sCTLA4, sGITR, sHVEM, sBTLA, sLAG3), showed an increase of the values of all the cytokines and chemokines. Patients in cluster B showed a significantly shorter PFS (3.5 months vs. 11.9 months in cluster A, p < 0.01), as shown by the Kaplan Meier curves in **Figure 4**. At the same time, PD was found in 94.4% of cluster B vs. 55.2% of cluster A patients (p = 0.04). Consequently, risk of PD was about 7 times higher in cluster B patients than in cluster A ones throughout anti-PD-1 treatment (Odd Ratio = 6.9, 95% C.I.(1.34-35.52)). Accordingly, SD was observed more often in Cluster A patients (34.5%) than in Cluster B ones (0%, OR = 0.1, 95% C.I.(0.01-0.91), p<0.05) (**Figure 3**).

## 4 Discussion

The challenge of immuno-oncology is the identification of new therapeutic strategies to overcome resistance to immunotherapy. Soluble immune profiles (SIP), resulting from the combined evaluation of circulating checkpoints, adhesion and inflammatory molecules (cytokines and chemokines) could be considered as a portrait of the immune system fitness of a patient, which may interfere or affect the response to treatment with ICIs. This study highlighted that given the variability of immune status, the analysis of circulating factors could provide meaningful prognostic and predictive information.

Mean basal values of soluble molecules differed according to tumor histology, suggesting that these differences may reflect a different organ-dependent immunity. In the examined patient cohort, NSCLC was characterized by a high expression of sICs, such as sCD27, sCD137, sHVEM and sLAG3, and by higher values of circulating cytokines and chemokines. On the other hand, HNSCCs presented the highest values of sPDL2, sCD80, sCTLA4, soluble adhesion molecules such as sICAM-1, sP-selectin and sE-selectin, while UMs showed the lowest values of cytokines and chemokines compared to NSCLCs and HNSCCs. Pre-treatment levels of several circulating molecules, regardless of tumor type, were associated with OS and PFS. Longer OS was reported in patients with low levels of sCD28, sGITR, sPDL1, sTIM3, IFNα, IFNγ, IL1β, IL10, IL1α, IL12p70, IL13, MIP1β and TNFα. Patients with OS of less than 12 months had significantly higher levels of multiple cytokines and chemokines: IFN α, IFNγ, IL1α, IL1β, IL10, IL12p70, IL13, TNFα and IL4.

In the landscape of soluble immune biomarkers, sICIs seem to be particularly promising, even though their predictive and prognostic meaning is still unclear and their role seems to depend on histology and on the setting of disease (13–15, 26).

In particular, sLAG3 (higher in NSCLC compared to UM and RCC) could be considered a marker of Th1 activation and DCs maturation, while high levels of sCTLA4 are associated with worse prognosis in patients affected by HNSCC, NSCLC, RCC and in colorectal cancer (CRC) (13, 14, 26–29). Furthermore, sPDL1 could contribute to the immune evasion mechanism, treatment resistance, and worse prognosis as well as sTIM3 values below the median (7972 pg/ml), reflecting the role attributed to their transmembrane form when expressed by tumor cells (18, 28–37).

Considering other soluble molecules and in accordance to the available literature, this study seems to show an association between IL10, IL13, IL1α, IL1β, TNFα and longer OS and PFS in several diseases (38–42). While the role of IFNγ in cancer is still controversial, this cytokine could exploit both anti-tumor and pro-tumor activities as well (43, 44). Lower IFNγ values at the baseline were reported in patients with squamous esophageal carcinoma responding to immunotherapies and in patients with RCC responding to the anti-VEGFR TKI (28, 39).

On the other hand, concentrations of IL4, MIP1α/β, IP10 and IL8 are significantly higher in subjects with lower OS, probably because they are involved in the processes of Th1 lymphocyte inhibition, induction of M2 differentiation in macrophages and in metastatic liver spreading (41, 44–50). The chaotic pattern of circulating cytokines can be interpreted through the identification of a ‘cytokines signature’, in which it is not the single cytokine which acquires a predictive value for response to immunotherapy, but the specific combination of several cytokines. This innovative approach has recently been explored in the literature with promising results (51–53).

**Figure 5:**
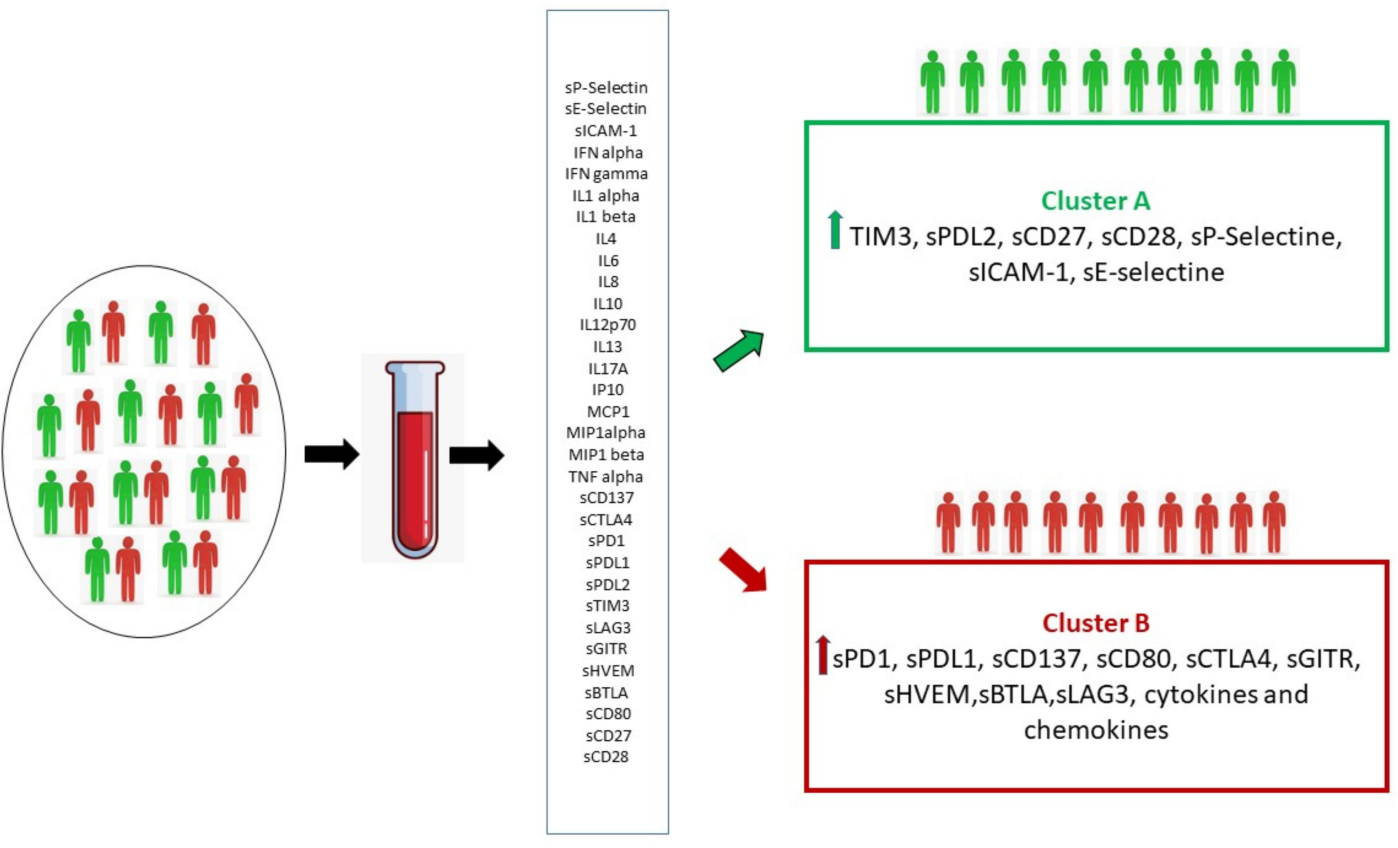
By means of cluster analysis, the circulating immune profile detected at baseline made it possible to identify two distinct groups of patients (Cluster A and B) characterized by the activation of different immune pathways resulting in two distinct clinical outcomes.

This portrait of the immune system emphasizes the complexity of molecules and solubility interactions and the difficulty of interpreting it, highlighting the need of an immunological comprehensive profile rather than the evaluation of individual markers. This study focused on the analysis of a soluble immune profile in relation to cancer outcomes. Two distinct groups of cancer patients were identified by means of cluster analysis, which take into account the pattern of soluble molecules detected at the baseline (**Figure 5**).

The first group was identified as the soluble immune profile (SIP) one which benefits the most, in terms of PFS and response, from immunotherapy (Cluster A). It seems probable that in this cluster the effect of the elevated value of the soluble checkpoints CD27 and CD80, which drive the differentiation of T-cells into memory cells, is preponderant (54). Thus, in this group the inhibitory activity of TIM3 and PD-L2 receptors is overcome by the cooperation of immune activating pathways and by immunotherapy (55).

In the second group (Cluster B), in addition to the numerical supremacy of inhibitory immune checkpoints, a high pro-inflammatory state is evident. The overexpression of the inhibitory PD-1/PD-L1, BTLA/HVEM, CTLA-4, LAG3 checkpoints axis, associated with the concurrent overelevation of cytokines and chemokines could play a decisive role in reducing the benefits obtainable with immunotherapy (55–59). Cytokines and chemokines have contrasting roles in promoting tumor immunity, inflammation, and response to immunotherapy. The presence of high levels of cytokines/chemokines in cluster B suggests an inflammatory state not capable of eliciting an active antitumor immune response. We know, to date, that elevated levels of some cytokines, such as IL6, are associated with worse outcomes to immunotherapy. However, in the inflammatory picture described in Cluster B, it is difficult to say which cytokine determines the pathway activation with dominant effect (60). The profound dysregulation of immune mechanisms, results in a hostile environment for the proper functioning of therapy with anti-PD-1 monoclonal antibodies. This soluble profile correlates to a significantly shorter PFS (3.5 versus 11.9 months).

The soluble profile varies widely by primary tumor type, as evidenced by their distribution in the two clusters. Cluster A collects mainly RCCs and NSCLCs, whereas Cluster B is dominated by HNSCCs. This finding confirms that important mechanisms of immunosuppression are involved in tumor progression of HNSCC, which could limit the efficacy of immunotherapy outside of combination strategies (61). All HNSCC patients included in the study were platinum refractory, representing a patient population achieving lower response rates to immunotherapy than the ones with tumor histotypes (62). Therefore, it is not surprising that the majority of patients with platinum refractory HNSCC had an unfavorable immune profile and fell into cluster B. However, it is relevant to note that through cluster analysis it was possible to identify a common immunological profile in non-responder patients. Presumably any patient falling into cluster B will have an unfavorable immune fitness and a tumor with an immunological behavior much more similar to that of a platinum-refractory HNSCC than to what would be expected on the basis of cancer type. The two clusters appear to be homogeneous in terms of rate of patients in the II-line and platinum-refractory settings, although the rate of platinum-refractory patients in cluster A is lower than cluster B (48.2 vs 57.1%, respectively). Recently, the possible immunomodulatory effect of chemotherapies has been studied to define the rationale of new combination strategies. Chemotherapeutic agents have different immunologic effects that could influence the response to immunotherapy. Cisplatin seems to be able to increase the activation and proliferation of T cells and their cytotoxic activity (63). In addition, recent *in vitro* and *in vivo* experiments have shown that cisplatin can enhance tumor immunogenicity by increasing MHC I cell surface expression, but, at the same time, it can induce up-regulation of PD-L1 in human and mouse ovarian cancer cell lines (64). Therefore, the role of prior treatments should be studied specifically and on larger, homogeneous populations in order to define the effect they may have on the soluble immune profile and outcomes to immunotherapy. Otherwise, RCCs, which are more represented in cluster A, had better outcomes, especially in terms of OS. In our population, RCCs had a mOS of 49.2 ± 20.7 months, higher than survival rates reported in the Checkmate 025 trial (65). However, in this series RCC patients had a favorable MSKCC risk. In addition, most of our patients with RCC had less than 2 sites of metastasis. Escudier et al. tried to investigate which baseline clinical factor was associated with better OS with nivolumab. In patients with 1 site of metastasis at baseline and a favorable MSKCC risk, OS was not reached at a median follow up of 22 months (66). In our series mUM patients are similarly distributed between clusters A and B. To date, no data are available from controlled clinical trials regarding immunotherapy in uveal melanoma, which in clinical practice is commonly treated in a similar fashion as cutaneous melanoma. However, recent prospective data have shown that the small proportion of patients who respond achieves significant disease control. Therefore, it is crucial to identify predictive factors for response (67).

The main limitation of this study is due to the small sample of patients involved and the heterogeneity of the population in terms of primary tumors, treatment line and patient prognosis. However, it provides important insights which should direct further investigation in a larger patient population. Surely this study could most likely be considered as a hypothesis generator, which should be validated on a more homogeneous population in terms of both histotype and treatment setting.

## 5 Conclusions

In conclusion, this study highlights: 1) a significant variability of immune status biomarkers in each patient; 2) an organ dependent immunity; 3) a significant association between multiple soluble ICs, cytokines/chemokines and outcome regardless of tumor type; 4) two soluble immune profiles, resulting from the combination of several circulating molecules, significantly associated with both treatment response and PFS. A predictive biomarker profile of oncological outcomes represents an urgent yet unmet need for a rational treatment of each patient based on their own immune features.

## Data availability statement

The datasets used and/or analyzed during the current study are available from the corresponding author on reasonable request.

## Ethics statement

The studies involving human participants were reviewed and approved by Ethical Committee no. 4421, “Sapienza University. The patients/participants provided their written informed consent to participate in this study.

## Author contributions

Conceptualization, AB, SM and PM; Data curation, AC, AC; SS and SP; Formal analysis, AS; Investigation, IGZ, ADF and MN; Resources, IGZ, ADF, MN; Supervision, ST, EC, MN, PM; Writing – original draft, GP, AB and SM; Writing – review & editing, BC, SA, ER, GS, GT. All authors contributed to the article and approved the submitted version.

## Funding

This research was funded by Sapienza University of Rome.

## Conflict of interest

PM has/had a consultant/advisory role for BMS, Roche, Genentech, MSD, Novartis, Amgen, Merck Serono, Pierre Fabre, and Incyte.

The remaining authors declare that the research was conducted in the absence of any commercial or financial relationships that could be construed as a potential conflict of interest.

## Publisher’s note

All claims expressed in this article are solely those of the authors and do not necessarily represent those of their affiliated organizations, or those of the publisher, the editors and the reviewers. Any product that may be evaluated in this article, or claim that may be made by its manufacturer, is not guaranteed or endorsed by the publisher.
