# Supplementary material for: The role of immune profile in predicting outcomes in cancer patients treated with immunotherapy
Source: Front Immunol. 2022 Nov 3;13:974087. doi: 10.3389/fimmu.2022.974087 (PMC9671166; doi:10.3389/fimmu.2022.974087)
Supplement: Supplementary file 1 [file DataSheet_1.docx]

**Supplementary Material**

**Table S1.** Mean values of each ICs in different subgroups and their comparison between pairs of tumor subgroups

|  | UM | | | RCC | | | HNSCC | | NSCLC | |  |
| --- | --- | --- | --- | --- | --- | --- | --- | --- | --- | --- | --- |
|  | *Mean±STD* | | | *Mean±STD* | | | *Mean±STD* | | *Mean±STD* | |  |
| BTLA | 1545.0±1154.7 | | | 1518.9±930.4 | | | 1851.3±702.9 | | 2629.0±3621.7 | |  |
| CD137 | 131.0±181.7 | | | 171.6±197.6 | | | 106.8±89.1 | | 1028.5±2750.9 | |  |
| CD27 | 4398.9±2994.7 | | | 10284.7±16291.3 | | | 6869.4±5450.8 | | 28588.9±25969.9 | |  |
| CD28 | 402.2±356.2 | | | 12611.7±35802.5 | | | 827.4±379.1 | | 6089.8±28924.7 | |  |
| CD80 | 480.2±525.7 | | | 514.4±902.2 | | | 1039.1±399.0 | | 382.3±916.9 | |  |
| CTLA4 | 196.5±139.6 | | | 267.1±349.6 | | | 389.8±152.7 | | 102.0±208.8 | |  |
| GITR | 56.7±96.4 | | | 288.1±547.0 | | | 19.0±18.2 | | 433.7±2056.3 | |  |
| HEVM | 76.7±125.5 | | | 1059.6±1182.1 | | | 11.2±16.5 | | 435.4±465.8 | |  |
| LAG3 | 253.6±142.1 | | | 304.3±235.1 | | | 508.4±357.2 | | 2776.5±12262.7 | |  |
| PD1 | 55.4±57.9 | | | 422.6±1121.6 | | | 127.0±93.7 | | 177.5±622.3 | |  |
| PDL1 | 15.9±15.5 | | | 2495.7±4376.6 | | | 2.0±.0 | | 38.4±52.4 | |  |
| PDL2 | 4326.6±3714.7 | | | 692.2±312.4 | | | 10213.2±3431.9 | | 153656.2±764092.7 | |  |
| TIM3 | 2322.7±2359.3 | | | 89623.6±263757.6 | | | 5572.7±3583.2 | | 9613.3±10980.1 | |  |
| *Mann-Whitney Test* | | | | | | | | | | | |
|  | | ***UM***  ***vs RCC*** | ***UM***  ***vs HNSCC*** | | ***UM***  ***vs NSCLC*** | ***RCC***  ***vs HNSCC*** | | ***RCC***  ***vs NSCLC*** | | ***HNSCC***  ***vs NSCLC*** | |
| BTLA | |  |  | |  |  | |  | |  | |
| CD137 | |  |  | | 0.005 |  | |  | | 0.012 | |
| CD27 | |  |  | | <0.001 |  | | <0.001 | | <0.001 | |
| CD28 | |  | 0.005 | | <0.001 |  | |  | |  | |
| CD80 | |  | 0.002 | |  | 0.008 | |  | | <0.001 | |
| CTLA4 | |  | 0.002 | | 0.001 | 0.049 | | 0.049 | | <0.001 | |
| GITR | |  |  | |  |  | |  | |  | |
| HEVM | | 0.012 | 0.006 | | <0.001 | <0.001 | |  | | <0.001 | |
| LAG3 | |  | 0.036 | | <0.001 |  | | 0.001 | |  | |
| PD1 | |  | 0.012 | |  | 0.041 | |  | | 0.008 | |
| PDL1 | |  | <0.001 | |  | <0.001 | |  | | 0.022 | |
| PDL2 | | <0.001 | 0.001 | | <0.001 | <0.001 | | <0.001 | |  | |
| TIM3 | |  | 0.004 | | <0.001 |  | | 0.002 | |  | |

UM uveal melanoma. RCC renal cell carcinoma. HNSCC head and neck squamous cell carcinoma. NSCLC non small cell lung cancer

**Table S2.** Mean values of each soluble adhesion molecules and comparison between different tumors subgroups.

|  | UM | | | RCC | | | | | HNSCC | | | NSCLC | |
| --- | --- | --- | --- | --- | --- | --- | --- | --- | --- | --- | --- | --- | --- |
|  | *Mean±STD* | | | *Mean±STD* | | | | | *Mean±STD* | | | *Mean±STD* | |
| E-selectin | 123299.3±133601.7 | | | 50287.5±9492.6 | | | | | 357640.9±105921.7 | | | 300838.6±52170.3 | |
| IcamI | 133369.5±132927.2 | | | 418248.2±518421.4 | | | | | 1073969.1±253473.6 | | | 367189.8±91726.6 | |
| P-selectin | 2256986.2±3588068.8 | | | 458484.7±611496.4 | | | | | 6809069.2±2750391.8 | | | 1325898.7±367449.1 | |
| *Mann-Whitney Test* | | | | | | | | | | | | | |
|  | ***UM vs RCC*** | | ***UM vs***  ***HNSCC*** | | | ***UM vs NSCLC*** | | ***RCC vs HNSCC*** | | | ***RCC vs NSCLC*** | | ***HNSCC vs NSCLC*** |
| E-selectin |  | <0.001 | | | 0.001 | | <0.001 | | | <0.001 | |  | |
| IcamI | 0.041 | <0.001 | | | <0.001 | | 0.002 | | |  | | <0.001 | |
| P-selectin |  | 0.001 | | |  | | <0.001 | | | 0.003 | | <0.001 | |

UM uveal melanoma. RCC renal cell carcinoma. HNSCC head and neck squamous cell carcinoma. NSCLC non small cell lung cancer

**Table S3.** Mean values of cytokines and chemokines in each cancer type and significant differences in serum levels between subgroups.

|  | | UM | | RCC | | | HNSCC | | NSCLC | |
| --- | --- | --- | --- | --- | --- | --- | --- | --- | --- | --- |
|  | | *Mean±STD* | | *Mean±STD* | | | *Mean±STD* | | *Mean±STD* | |
| IFNα | | 22.2±43.0 | | 17.0±22.5 | | | 24.2±18.0 | | 91.5±57.0 | |
| IFNγ | | 147.0±185.4 | | 182.1±258.9 | | | 302.1±170.2 | | 1200.9±531.9 | |
| IL1β | | 23.3±52.0 | | 25.0±53.7 | | | 28.1±24.7 | | 207.3±218.2 | |
| IL10 | | 7.1±8.3 | | 26.2±58.7 | | | 17.3±1.1 | | 412.0±74.5 | |
| IL1α | | 137.1±519.6 | | 47.8±57.5 | | | 21.0±32.5 | | 376.7±200.1 | |
| IL12p70 | | 194.7±118.4 | | 251.2±200.2 | | | 202.8±130.5 | | 881.7±451.7 | |
| IL13 | | 13.2±16.6 | | 22.6±26.8 | | | 15.5±18.1 | | 198.0±203.5 | |
| IL17A | | 85.4±116.8 | | 98.9±117.9 | | | 150.5±80.8 | | 96.4±35.8 | |
| IL4 | | 138.7±199.4 | | 356.0±527.7 | | | 177.8±100.6 | | 2351.1±1099.7 | |
| IL6 | | 93.0±230.5 | | 435.1±749.5 | | | 167.8±144.1 | | 1120.3±1175.7 | |
| IL8 | | 98.4±239.9 | | 381.1±745.2 | | | 333.3±519.4 | | 1066.7±1646.4 | |
| IP10 | | 177.7±166.4 | | 403.4±337.6 | | | 579.6±583.5 | | 1878.3±924.8 | |
| MCP1 | | 805.2±1345.6 | | 233.9±127.3 | | | 1455.3±645.7 | | 3223.1±1379.1 | |
| MIP1α | | 62.7±109.7 | | 58.8±34.8 | | | 104.8±61.7 | | 883.7±273.7 | |
| MIP1β | | 628.1±1359.3 | | 284.3±310.7 | | | 839.4±511.4 | | 3732.7±1177.5 | |
| TNFα | | 795.8±1093.0 | | 283.0±221.7 | | | 1774.6±1003.1 | | 2968.72638 | |
|  | ***UM vs RCC*** | | ***UM vs HNSCC*** | | ***UM vs NSCLC*** | ***RCC vs HNSCC*** | | ***RCC vs NSCLC*** | | ***HNSCC vs NSCLC*** |
| IFNα |  | | 0.029 | | <0.001 |  | | <0.001 | | 0.002 |
| IFNγ |  | | 0.006 | | <0.001 | 0.013 | | <0.001 | | <0.001 |
| IL1β |  | | 0.022 | | <0.001 |  | | <0.001 | | <0.001 |
| IL10 |  | | 0.001 | | <0.001 | 0.008 | | <0.001 | | <0.001 |
| IL1α |  | |  | | <0.001 |  | | <0.001 | | <0.001 |
| IL12p70 |  | |  | | <0.001 |  | | <0.001 | | <0.001 |
| IL13 |  | |  | | <0.001 |  | | <0.001 | | <0.001 |
| IL17A |  | | 0.005 | | 0.028 | 0.03 | |  | | 0.025 |
| IL4 |  | | 0.048 | | <0.001 |  | | <0.001 | | <0.001 |
| IL6 |  | | 0.002 | | <0.001 |  | | 0.018 | | <0.001 |
| IL8 |  | | 0.01 | | <0.001 |  | | 0.015 | | 0.012 |
| IP10 | 0.01 | | 0.001 | | <0.001 |  | | <0.001 | | <0.001 |
| MCP1 |  | | 0.002 | | <0.001 | <0.001 | | <0.001 | | 0.001 |
| MIP1α |  | | 0.007 | | <0.001 |  | | <0.001 | | <0.001 |
| MIP1β |  | | 0.006 | | <0.001 | 0.004 | | <0.001 | | <0.001 |
| TNFα |  | | 0.007 | | <0.001 | <0.001 | | <0.001 | | 0.001 |

UM uveal melanoma. RCC renal cell carcinoma. HNSCC head and neck squamous cell carcinoma. NSCLC non small cell lung cancer

**Table S4.** Correlation between serum cytokines and chemokines levels and long surviving patients (OS > 12 months).

|  | **OS** | **N°** | **Mean** | **Std. Deviation** | **Std. Error Mean** | ***p>0.05*** |
| --- | --- | --- | --- | --- | --- | --- |
| **IFN α** | < 12  >12 | 19  33 | 70.0  24.4 | 66.8  32.3 | 15.3  5.6 | *P = 0.008* |
| **IFN γ** | < 12  >12 | 19  33 | 792.3  310.9 | 679.9  382.4 | 155.9  66.6 | *P = 0.008* |
| **IL10** | < 12  >12 | 19  33 | 209.2  70.7 | 221.5  138.4 | 50.8  24.1 | *P = 0.008* |
| **IL12p70** | < 12  >12 | 19  33 | 612.3  267.1 | 526.3  235.9 | 120.7  41.1 | *P = 0.009* |
| **IL13** | < 12  >12 | 19  33 | 125.7  31.1 | 205.2  47.4 | 47.1  8.2 | *P = 0.023* |
| **IL1 α** | < 12  >12 | 19  33 | 219.5  128.8 | 213.8  429.0 | 49.0  74.7 | *P = 0.007* |
| **IL1 β** | < 12  >12 | 19  33 | 116.7  34.6 | 152.3  45.2 | 34.9  7.9 | *P = 0.035* |
| **IL4** | <12  >12 | 19  33 | 1528.1  391.1 | 1535.1  610.5 | 352.2  106.3 | *P = 0.008* |
| **IL8** | <12  >12 | 19  33 | 506.8  269.9 | 497.1  522.9 | 114.0  91.0 | *P = 0.019* |
| **MCP1** | < 12  >12 | 19  33 | 2104.4  1244.2 | 1656.8  1469.9 | 380.1  255.9 | *P = 0.021* |
| **MIP1 α** | < 12  >12 | 19  33 | 484.3  165.1 | 420.6  253.4 | 96.5  44.1 | *P = 0.008* |
| **MIP1 β** | < 12  >12 | 19  33 | 2143.9  1046.6 | 1775.5  1492.4 | 407.3  259.8 | *P = 0.033* |
| **TNF α** | < 12  >12 | 19  33 | 2210.1  1230.2 | 1397.8  1182.7 | 320.7  205.9 | *P = 0.018* |

**Table S5.** **Correlation between serum value of ICs, cytokines and chemokines and OS.**

| **Soluble factor (median)** |  | **N°** | **Mean OS ± Std** | **Significance** |
| --- | --- | --- | --- | --- |
| **sCD28**  **(768)** | Above median | 29 | 25.7 ± 4.6 | *P = 0.002* |
|  | Below median | 35 | 54.9 ± 7.9 |  |
| **sGITR**  **(47)** | Above median | 31 | 29.3 ± 4.8 | *P = 0.027* |
|  | Below median | 31 | 57.9 ± 9.4 |  |
| **sPD-L1**  **(15)** | Above median | 31 | 26.5 ± 4.2 | *P = 0.001* |
|  | Below median | 33 | 58.9 ± 8.7 |  |
| **sTIM3**  **(7972)** | Above median | 32 | 31.9 ± 6.5 | *P = 0.029* |
|  | Below median | 32 | 48.7 ± 7.2 |  |
| **IFN α**  **(93.6)** | Above median | 19 | 27.9 ± 7.0 | *P = 0.041* |
|  | Below median | 25 | 44.5 ± 7.8 |  |
| **IFN γ**  **(1213)** | Above median | 24 | 23.5 ± 5.4 | *P = 0.002* |
|  | Below median | 20 | 51.9 ± 8.9 |  |
| **IL1 β**  **(97.8)** | Above median | 24 | 23.5 ± 5.4 | *P = 0.002* |
|  | Below median | 20 | 51.9 ± 8.9 |  |
| **IL10**  **(399.5)** | Above median | 15 | 21.5 ± 5.9 | *P = 0.008* |
|  | Below median | 29 | 46.6 ± 7.9 |  |
| **IL1 α**  **(300)** | Above median | 24 | 20.1 ± 3.6 | *P = 0.002* |
|  | Below median | 20 | 51.4 ± 8.6 |  |
| **IL12p70**  **(885.7)** | Above median | 25 | 28.3 ± 5.9 | *P = 0.042* |
|  | Below median | 19 | 52.2 ± 10.0 |  |
| **IL13**  **(90)** | Above median | 22 | 23.5 ± 5.5 | *P = 0.016* |
|  | Below median | 22 | 55.3 ± 9.9 |  |
| **MIP1 β**  **(3595)** | Above median | 21 | 20.7 ± 3.9 | *P = 0.006* |
|  | Below median | 23 | 48.4 ± 8.3 |  |
| **TNF α**  **(2896.3)** | Above median | 25 | 32.5 ± 8.3 | *P = 0.044* |
|  | Below median | 19 | 44.4 ± 7.3 |  |

**Table S6.** Correlation between serum value of sICs. cytokines and chemokines and PFS.

| **Soluble factor (median)** |  | **N°** | **Mean PFS ± Std** | **Significance** |
| --- | --- | --- | --- | --- |
| **sCD28**  **(768)** | Above median | 29 | 11.2 ± 2.9 | *P = 0.016* |
|  | Below median | 34 | 25.9 ± 4.4 |  |
| **sGITR**  **(47)** | Above median | 31 | 11.5 ± 2.8 | *P = 0.016* |
|  | Below median | 30 | 21.7 ± 3.4 |  |
| **sPD-L1**  **(15)** | Above median | 31 | 11.1 ± 2.7 | *P = 0.006* |
|  | Below median | 32 | 27.9 ± 4.6 |  |
| **IL10**  **(399.5)** | Above median | 15 | 5.8 ± 1.8 | *P = 0.019* |
|  | Below median | 28 | 17.0 ± 3.2 |  |
| **IL13**  **(90)** | Above median | 22 | 6.0 ± 1.4 | *P = 0.007* |
|  | Below median | 21 | 19.8 ± 3.9 |  |
